# The double burden of disease of COVID-19 in cardiovascular patients: overlapping conditions could lead to overlapping treatments

**DOI:** 10.1007/s10654-020-00628-1

**Published:** 2020-04-15

**Authors:** Nathalia Gonzalez-Jaramillo, Nicola Low, Oscar H. Franco

**Affiliations:** 1 Institute of Social and Preventive Medicine, University of Bern, Mittelstrasse 43, 3012 Bern, Switzerland; 2 Graduate School of Health Sciences, University of Bern, Bern, Switzerland

Coronavirus disease 2019 (COVID-19) pandemic imposes a double burden on people with cardiovascular disease (CVD). About 40% of hospitalized COVID-19 patients have CVD [1] and the clinical course of COVID-19 is more severe in patients with hypertension, diabetes, and CVD [2]. COVID-19 mortality increases with comorbidities and age [3]. Correct evaluation of this double burden is challenged by three gaps in our knowledge: (1) the unknown confounding effect of age on mortality of CVD/COVID-19 patients, (2) the not fully understood effects of COVID-19 on the cardiovascular system, and (3) how antihypertensive medications targeting the renin–angiotensin system (RAS) might be associated with the severity of and survival to COVID-19 in CVD patients.

COVID-19 is caused by a new human coronavirus, severe acute respiratory syndrome coronavirus 2 (SARS-CoV-2) first identified in Wuhan, China. Early estimates of the basic reproduction number were around 2.2 (90% high-density interval 1.4–3.8) [4], and as of 11 April 2020, there were over 1,720,000 confirmed cases of COVID-19 and more than 100,000 deaths. Novel human coronaviruses have caused two other pandemics in less than two decades: the first severe acute respiratory syndrome (SARS) coronavirus began in late 2002 and caused around 1000 deaths. Middle East Respiratory Syndrome (MERS), first identified in 2012, has killed 862 persons so far. Due to the lack of pre-existing immunity to the new virus, it is reasonable to believe that it may infect everyone in a given population equally. However, the majority of people with COVID-19 are older than 40 years [1], as are the majority of people with CVD.

**Fig. 1:**
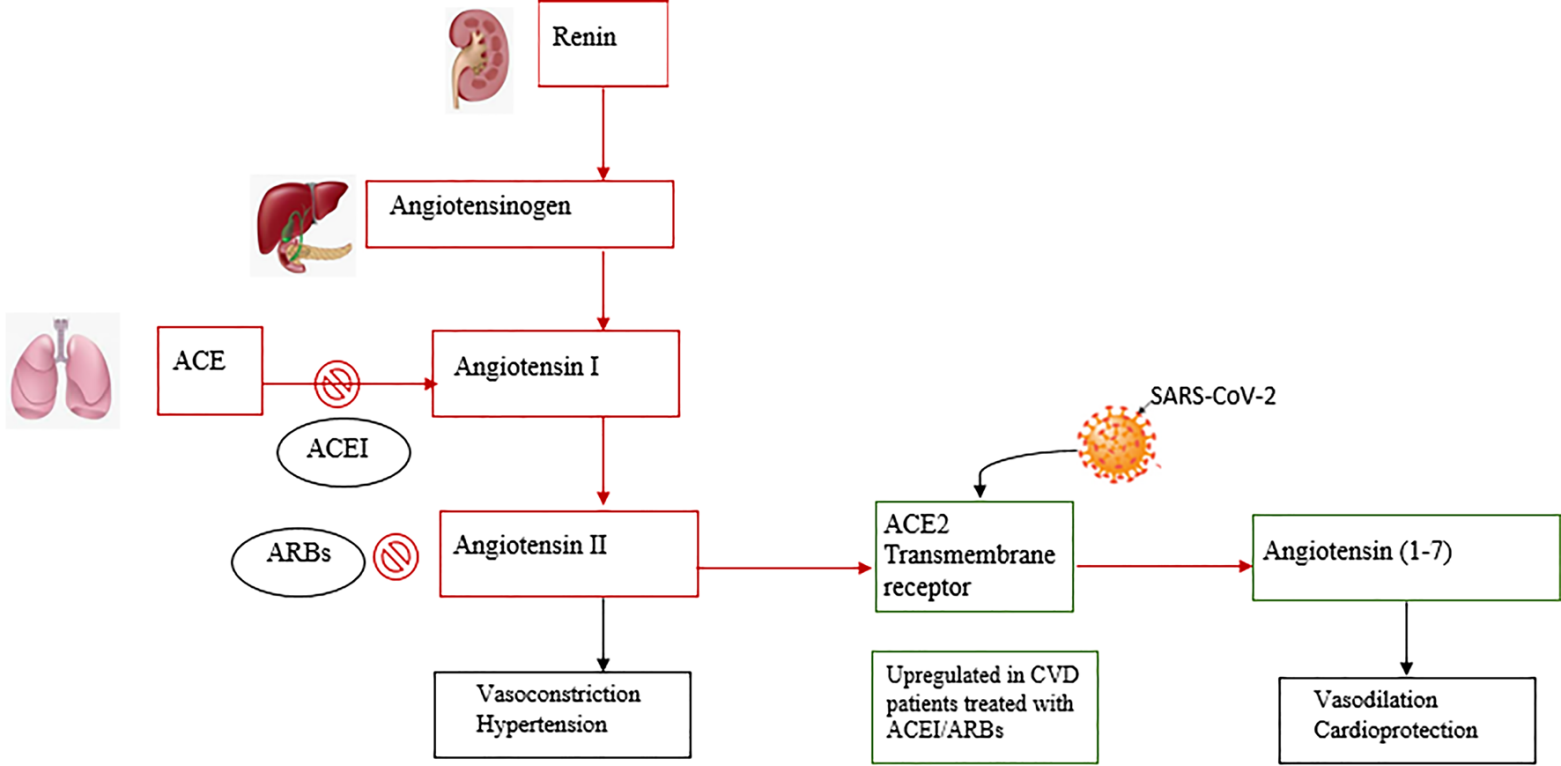
Renin–angiotensin system inhibition (RAS) by Angiotensin-converting enzyme/Ang-II receptor blockers (ACEI/ARBs) and SARS-CoV-2 binding to ACE2 receptors. Red squares indicate RAS actions, green squares indicate ACE actions. Black squares indicate effects for each component

Soon after the discovery of SARS-CoV-2, researchers found that virus entry into the cell is initiated by binding to the angiotensin-converting enzyme 2 receptor (ACE2), a component of RAS [5]. RAS is a complex network mediating cardiovascular and renal function (Fig. 1). It plays a crucial role in the endocrine regulation of fluid volume and electrolytes. Pathological RAS hyperactivation is associated with acute cardiac, pulmonary, and renal damage [6]. RAS has also been implicated in inflammation, proliferation, and fibrosis in chronic pulmonary diseases such as idiopathic pulmonary fibrosis and pulmonary arterial hypertension [7]. The ACE2 receptor is an integral membrane protein highly expressed in the lungs and heart that physiologically counteracts the activity of RAS by converting the vasoconstrictor angiotensin-II (Ang-II) to the vasodilator angiotensin-(1–7). Evidence from experimental studies indicates that a decrease in ACE2 is associated with the progression of type 2 diabetes mellitus [8] and myocardial hypertrophy [9]. Conversely, upregulated ACE2 improves glycaemic control in diabetes [8], prevents myocardial fibrosis, and improves cardiac function after myocardial infarction [10]. Known protective effects of ACE2 through angiotensin-(1-7) might be attributable to antagonism of Ang-II signalling.

SARS-CoV-2 infection produces enzymatic shedding that inactivates ACE2 and prevents conversion of Ang-II [11]. This effect could in part explain the cardiovascular and respiratory manifestations of COVID-19. Following a decrease in ACE2, an increase in vasoactive, proliferative, and profibrotic Ang-II leads to cardiopulmonary damage through hemodynamic changes such as pulmonary hypertension and interstitial edema followed by respiratory failure in the most severe cases [12]. The use of soluble ACE2 to counteract pulmonary damage has been studied in two phase-II trials involving acute lung injury or acute respiratory distress syndrome, and pulmonary arterial hypertension. Soluble ACE2 has also been studied in a phase-I trial to assess its effects on hypoxia after exercise. In spite of its cardiometabolic protective function, ACE2 failed to demonstrate meaningful improvement in physiological or clinical measures in its pharmacological form [7, 13].

Effective interventions against SARS-CoV-2 need to be developed rapidly. Timely sequencing of the virus has already led to the development of several new molecular targets and vaccine candidates, but even with the most optimistic timetable for clinical trials and regulatory approval new products are not expected for around 18 months. Repurposing existing treatments is thus a potentially important short-term strategy to respond to COVID-19. Therefore, if proven protective in COVID/CVD patients, RAS inhibitors could potentially become a standard treatment for other COVID patients.

RAS inhibitors, ACE inhibitors (ACEI) and Ang-II receptor blockers (ARBs), are safe and efficacious in the treatment of patients with CVD. Their use to counteract the effects of the lack of function of the ACE2 protein in COVID-19 [14] seems more than logical and currently multiple specialty societies recommend that RAS inhibitors should be continued in patients who are currently under treatment [15]. However, both preclinical and clinical studies have shown that ACE2 is upregulated in patients under treatment with ACEI/ARBs [16]. An increase in receptor availability might facilitate SARS-CoV-2 replication, so this pharmacological approach might not be helpful for treating COVID-19 [17]. Therefore, beyond the theoretical pros and cons for using RAS inhibitors in this pandemic, there are three important research questions. First, is mortality in CVD patients a consequence of virus entry using ACE2 receptors that might improve with RAS blocking? Second, could the use of ACEI/ARBs enhance SARS-CoV-2 replication and increase the severity of COVID-19? If RAS blocking increases the risk of severe COVID-19 and mortality in CVD patients, clinicians may need to replace these drugs during treatment of infection. Third, could RAS inhibitors be used to treat other COVID patients? If found to be protective, RAS inhibitors may become standard-of-care therapies for this and future outbreaks. Current recommendations need to be based on the current pandemic evidence. A remarkable amount of knowledge about this new coronavirus has been gained in a short period of time. Now, rapid observational studies are needed to clarify possible associations and to evaluate treatment strategies to reduce the double burden of COVID-19 in CVD patients. Narrowing these knowledge gaps may help identify potential effective interventions against COVID-19 and closely related coronaviruses both in CVD and non-CVD patients.
